# Safety and feasibility of ultra-long construct navigated minimally invasive spine surgery with adjuvant radiotherapy in extensive spinal metastasis : a comparative analysis

**DOI:** 10.1186/s12885-023-11729-x

**Published:** 2023-12-18

**Authors:** Borriwat Santipas, Monchai Ruangchainikom, Sirichai Wilartratsami, Supachat Jiamamornrat, Nhathita Panatreswas, Panya Luksanapruksa

**Affiliations:** 1 Division of Spine Surgery, Department of Orthopedic Surgery, Department of Orthopedic Surgery, Faculty of Medicine Siriraj Hospital, Mahidol University, 2 Wanglang Road, Bangkok Noi, Bangkok, 10700 Thailand; 2 Research unit, Department of Orthopedic Surgery, Faculty of Medicine Siriraj Hospital, Mahidol University, Bangkok, Thailand

**Keywords:** Ultralong construction, Minimally invasive spine surgery, Radiotherapy, Multiple spinal metastasis, Extensive spinal metastasis

## Abstract

**Background:**

Our study compares the outcomes of extensive spinal metastasis patients treated with Ultra-Long Construct Navigated Minimally Invasive Spine Surgery (UNMISS) with Adjuvant Radiotherapy to those receiving only radiotherapy. Spinal metastasis often necessitates interventions like radiotherapy, chemotherapy, or surgery, with an increasing trend towards surgical management. minimally invasive spine surgery has demonstrated advantages over traditional open surgery, with fewer complications and better postoperative outcomes. Radiotherapy continues as a standard for those unsuitable for surgery.

**Methods:**

This retrospective study included extensive spinal metastasis patients treated between January 2017 and December 2020. We compared patients undergoing UNMISS in conjunction with radiotherapy to patients receiving radiotherapy alone, evaluating demographic data, disease characteristics, and treatment outcomes (VAS, survival) to establish statistical significance.

**Results:**

Twenty-three patients were included in our study. Fourteen patients underwent UNMISS, and nine patients received radiotherapy alone. There was no difference in baseline characteristics of patients. The longest construct in our case series involved T1 to iliac. Both cohorts showed significant improvement in pain scores post-treatment (*p* = 0.01). However, the UNMISS group demonstrated significantly lower post-treatment VAS scores (*p* = 0.003), indicating enhanced pain relief. Survival outcomes did not differ significantly between the two groups.

**Conclusion:**

The UNMISS should be considered as an alternative treatment in a patient with symptomatic extensive spinal metastasis. The primary goal of this technique is to stabilize the multiple levels of spinal metastasis and decompression of the neural element if needed. This technique is safe and has a better outcome in pain improvement than the patient who received radiotherapy alone.

## Background

Spinal metastasis is common in cancer patients and accounts for almost 70% of skeletal metastases [1]. Treatment of spinal metastasis involves radiotherapy, chemotherapy, and surgery. The incidence of these patients who require surgical intervention is increasing from time to time [2]. The neurological deficit, spinal instability, and the individual patient usually guide surgical management. The selected surgical intervention correlates with the extent of spinal metastasis lesion. Radical vertebrectomy or Tomita en bloc resection is rarely indicated and traditionally reserved for curative in the case of a single intracompartmental lesion [3]. Other surgical interventions included stabilizing involved segments with or without the decompression for the spinal cord of root decompression. The open surgery had been shown complication rates of up to 18%, which resulted from extensive blood loss and postoperative wound complications [4].

Patients with extensive spinal metastases (surgical classification of spinal tumors type 7) usually present with pain, instability, or neurological deficit and require palliative surgery. Previously, there were few reports on the treatment strategy of those patients with long construct spinal stabilization [5]. Minimal invasive spinal stabilization provides a good outcome as a palliative procedure in symptomatic spinal metastasis patients. There has been associated with a lower complication profile, shorter hospital stays, shorter operative time, less blood loss, and better functional outcomes when compared to open surgery [6–10]. Radiotherapy is the primary treatment for patients who are not surgical candidates, are denied surgical treatment, and limited life expectancy. And provide the satisfying outcome of pain relief and control of paralysis.

We defined the ultra-long construct navigated minimally invasive spine surgery (UNMISS) with more than ten levels of operated spinal segments. We compared the treatment outcomes of patients undergoing UNMISS with adjuvant radiotherapy at our tertiary referral center in terms of safety, pain reduction, and survival with those who received only radiotherapy.

## Methods

We performed a comparative, retrospective cohort study on extensive spinal metastasis patients. The data were obtained from the medical database of the Department of Orthopedic Surgery, Faculty of Medicine. We identified consecutive spinal metastasis patients who underwent palliative spinal surgery, which involved more than ten vertebral body levels, and the patients who were planned to undergo UNMISS but denied surgery and received radiotherapy alone between January 2017 and December 2020. The study was approved by the Institutional Review Board (IRB), Siriraj Institutional Review Board (SIRB) (COA No. 569/2563 (IRB1)).

Within our institution, the criteria for surgical intervention in spinal metastasis are predicated on a multidisciplinary approach. Candidates for surgery are those who present with intractable pain not alleviated by medication, spinal instability as quantified by the Spinal Instability Neoplastic Score (SINS) [11], or neurological deficits that necessitate both decompression and stabilization.

For the purpose of this study, patients with extensive spinal metastasis requiring fixation across more than ten vertebral levels were selected for the UNMISS procedure. This technique is specifically indicated for patients with widespread metastatic disease, where conventional methods would not suffice due to the extensive nature of the required stabilization.

In contrast, patients with less extensive spinal metastasis were managed using alternative surgical techniques, including open surgical methods, which are more suitable for shorter spans of spinal involvement. The determination of the appropriate surgical technique was made after thorough evaluation by our spine surgery team, taking into account the individual patient's disease characteristics, the extent of spinal involvement, and the potential benefits and risks of the procedure.

**Fig. 1:**
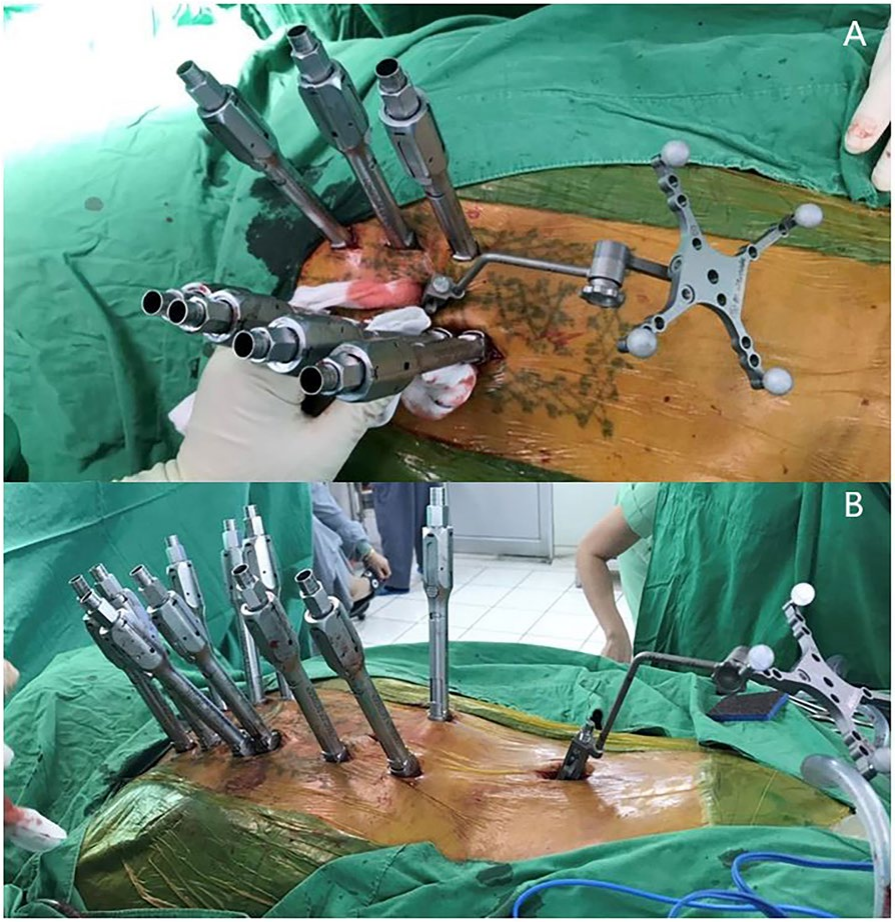
The navigated percutaneous pedicular screws fixations were usually required two times of spinal landmark registration due to the ultra-long of the operated segment. **A** The application of systems of thoracic region, **B** The application of systems of the lumbosacral region

**Fig. 2:**
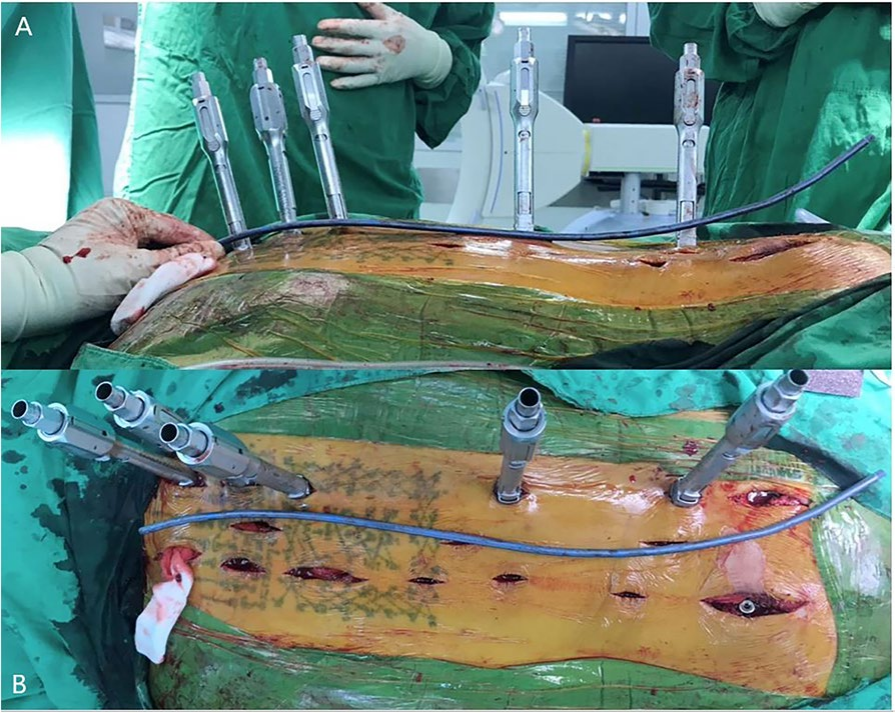
**A** Long whole soft rods were contoured into the physiologic sagittal curve before insertion. **B** The minimally invasive incision for instrumentation

**Fig. 3:**
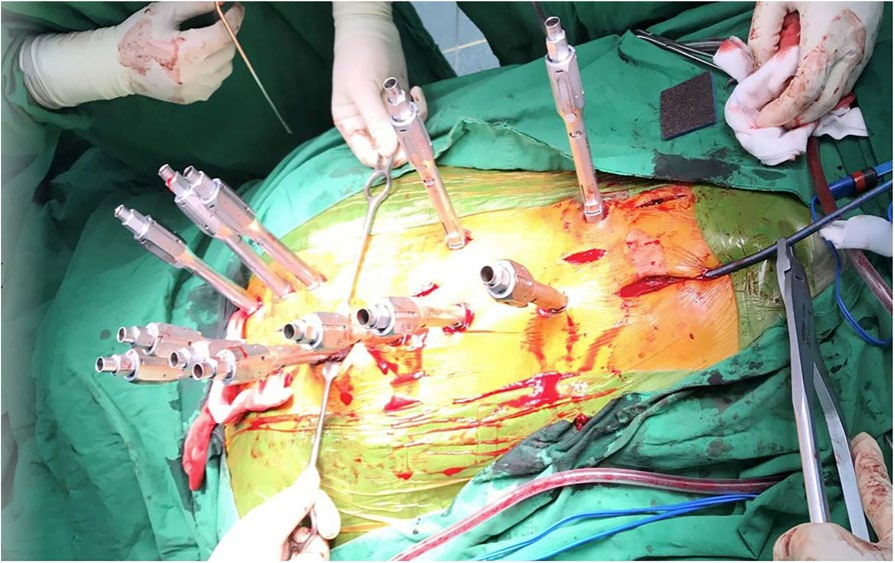
Contoured physiologic rods were inserted in the subfascial sheath from caudal to cranial by rotating 180 degrees when the uppermost of the rods pass through the thoracolumbar area, and then finely adjusted alignment with in situ rod bender was performed

**Fig. 4:**
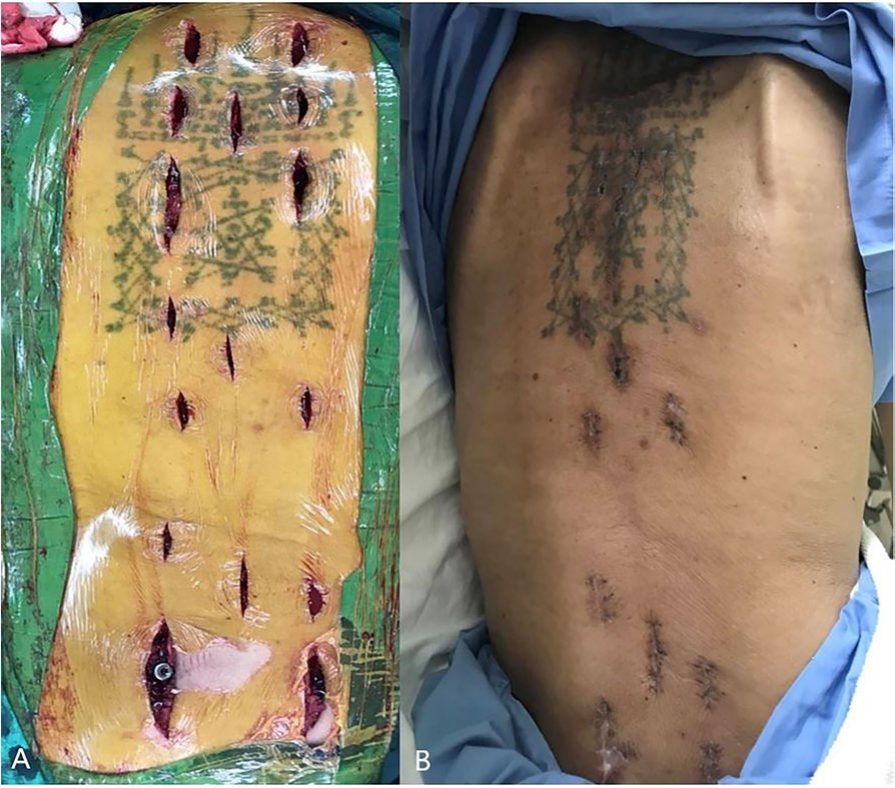
**A** A Mini-midline approach was performed for decompression in the case which required direct decompression. **B** The wound was completely healed after three weeks of operation without complication

**Fig. 5:**
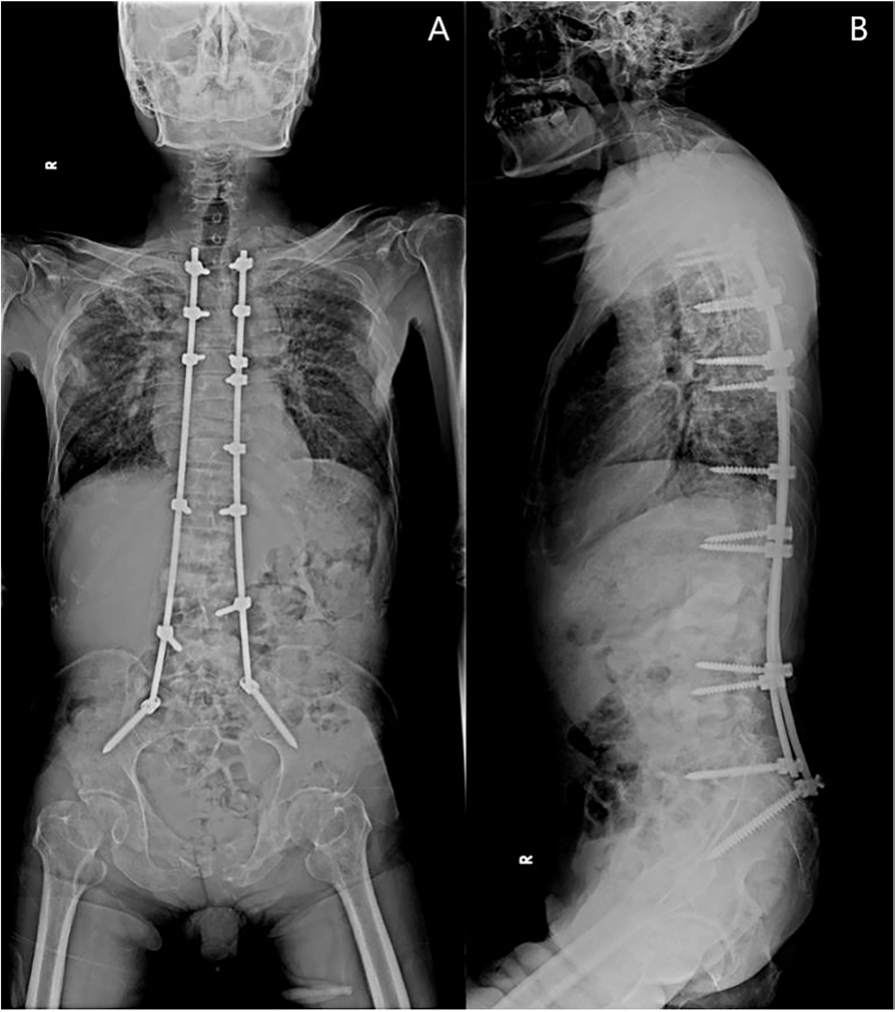
**A** Antero-posterior view of postoperative radiograph. **B** Lateral view of postoperative radiograph

Patients who received surgical treatment were operated on by two experienced fellowship-trained spine surgeons with the same surgical steps. We performed the minimally invasive approach for the instrumented level. The insertion of percutaneous pedicular screws (Medtronic Legacy pedicular screw and Longitude Extender system) under O-arm navigation. Navigated percutaneous pedicular screw fixations usually required two times of spinal landmark registration due to the ultra-long of the operated segment (Fig. 1A and B). Long whole soft rods were pre-contoured to match the normal sagittal profile (thoracic kyphosis and lumbar lordosis between 20–40 degrees) or maintained in-situ if no significant sagittal deformity was present before insertion (Fig. 2A and B). Pre-contoured rods were inserted in the subfascial sheath from caudal to cranial by rotating 180 degrees when the uppermost of the rods passed through the thoracolumbar area, and then finely adjusted alignment with in situ rod bender was performed (Fig. 3). Decompression via a mini-midline approach was specifically indicated for patients exhibiting neurological deficits or those with metastatic epidural spinal cord compression graded at 1C, 2, or 3 on MRI (Fig. 4A and B) [12]. To determine the levels necessitating decompression, we employed a detailed correlation between the neurological level of injury, as defined by the ASIA neurological classification [13], and the corresponding MRI findings. To ensure thorough decompression and alleviate spinal cord tension, the procedure was extended to one vertebral level above and below the index level. We did not perform anterior reconstruction or augmentation, or fusion surgery in our case series. Examples of postoperative radiographs are shown in Fig. 5A and B. All patients receive postoperative radiotherapy within two weeks after they completely wound healed. Patients who were advised to undergo UNMISS and denied surgery will receive radiotherapy alone. The radiotherapy protocol was same in both groups, which patients received 30 Gy in 10 fractions with the three-dimensional conformal radiotherapy (3D-CRT).

We collect the demographic data and characteristics of patients, including gender, age, primary tumor, New England Spinal Metastasis Score [14], Tomita score, pre-treatment and 30-day post-treatment Visual Analog Scale (VAS) scores, operative time, intraoperative blood loss, postoperative complication, and survival outcome.

All statistical analyses were conducted using SPSS ver. 18.0 software (SPSS Inc., Chicago, IL, USA). The Chi-square test and one-way ANOVA test were performed to evaluate the difference in each parameter. *P* values < 0.05 were considered significantly different.

## Results

We included 23 patients diagnosed with extensive spinal metastases (surgical classification of spinal tumors type 7) [15], who presented with symptomatic pathological fractures or neurological deficit from spinal cord compression.

Fourteen patients underwent minimally invasive palliative spine surgery under O-arm navigation. Eleven patients (78.5%) were female. The median age was 53.5 (Interquartile range = 50.5–60.75). Eight patients (57.14%) had primary breast cancer. Five patients (35.7%) had primary lung cancer. Ten patients experienced neurological deficits. The average VAS was 6.71.

Nine patients received radiotherapy alone. Seven patients (77.78%) were female. The median age was 66 (Interquartile range = 49–71). Four patients (44.44%) had primary breast cancer. Two patients (22.22%) had primary lung cancer. The average VAS was 7.66.

There were no statistical differences in gender, age, pre-operative VAS, primary tumor, Tomita score, and NESMS between the group. Details of patients' characteristics are shown in Tables 1, 2 and 3.

**Table 1:** Details of included individual patients of UNMISS group

No	Age	Sex	Primary	NESMS	Tomita	Pre-op Frankel	Post-op Frankel	Pre-op VAS	Post-op VAS	Decompression	Operated level	Operative time	Blood loss	Complication	Survival, months
1	69	F	Breast	1	3	D	D	8	0	Yes	T9 – iliac	335	420	No	12
2	53	F	Breast	0	7	D	D	10	2	No	T8-S2	190	350	No	3
3	62	F	Lung	1	10	E	D	10	0	Yes	**T1-iliac**	370	650	Motor weakness	4
4	57	M	Lung	1	10	C	C	7	0	Yes	T3-iliac	365	500	No	2
5	54	M	Renal	1	8	D	D	5	0	Yes	T10-S2	335	800	No	7
6	44	F	Breast	2	7	C	C	8	0	Yes	C2-T5	200	600	UTI	2
7	46	F	Breast	2	7	E	E	5	0	No	C2-T6	210	350	Wound dehiscence	7
8	65	F	Breast	3	3	D	E	7	0	Yes	C5-L2	175	400	No	40^a^
9	52	F	Breast	2	3	E	E	8	0	No	T1-S2	275	800	No	8
10	78	F	Lung	1	10	D	D	7	0	Yes	T1-T5, T12-L4	170	450	No	17
11	52	F	Breast	3	3	D	D	7	2	Yes	T1-S2	270	300	Wound dehiscence	25^a^
12	50	F	Breast	2	7	E	E	4	0	No	C5-T3, T8-L3	295	450	No	4^b^
13	56	M	Lung	0	8	C	C	6	0	Yes	T2-iliac	360	1400	No	10^b^
14	50	F	Lung	2	6	D	E	2	0	Yes	C7-L1	265	800	No	16^a^

^a^patient still alive

^b^loss to follow up

**Table 2:** Details of included individual patients of RT alone group

No	Age	Sex	Primary	NESMS	Tomita	Pre-op Frankel	Pre-RT VAS	Post-RT VAS	Survival, months
1	41	F	Thyroid	1	5	B	10	6	21^b^
2	64	M	Prostate	2	5	D	10	9	6^a^
3	66	F	Breast	1	10	D	8	6	18^a^
4	72	F	Breast	1	7	E	2	3	26^a^
5	45	M	Nasophatynx	1	10	B	10	5	3^b^
6	49	F	Breast	1	5	E	8	2	24^b^
7	69	F	Lung	2	8	E	10	5	18
8	71	F	Lung	1	10	D	9	8	15^b^
9	71	F	Breast	1	7	B	7	6	1^b^

^a^patient still alive

^b^loss to follow up

**Table 3:** Demographic data

Data	N (%) | Median (IQR) | Mean ± SD		*P*-value
	**UNMISS + RT (*N* = 14)**	**RT Alone (*N* = 9)**	
**Age (years)**	53.5 (50.5–60.75)	66 (49–71)	0.361
**Female**	11 (78.5)	7 (77.78)	0.639
**Primary tumor**			0.326
Breast cancer	8 (57.14)	4 (44.44)	
Lung cancer	5 (35.71)	2 (22.22)	
Renal cell carcinoma	1 (7.15)	-	
Thyroid	-	1 (11.11)	
Prostate	-	1 (11.11)	
Nasopharynx	-	1 (11.11)	
**NESMS**			0.198
(1 yr postop survival rate)			
0 (18.5%)	2 (14.30)	-	
1 (34.9%)	5 (35.71)	7 (77.78)	
2 (46.2%)	5 (35.71)	2 (22.22)	
3 (68.3%)	2 (14.30)	-	
**Tomita**			0.205
(Avg survival, months)			
2–4 (19 months)	4 (28.58)	-	
5–7 (16 months)	5 (35.71)	5 (55.56)	
8–10 (3 months)	5 (35.71)	4 (44.44)	
**Pre-treatment VAS**	6.71 ± 2.20	7.66 ± 3.01	0.439

### Surgically treated patients

The number of operated vertebral body range from 10 to 20 segments. The patient who underwent the most extended operated segment was diagnosed with primary lung cancer with spinal metastasis of T2 T10 L1 and L2 vertebral bodies. The operated segment included T1 to the iliac bone with fourteen pedicular screws. The median blood loss was 475 (405–762.5) ml. The average blood loss of the UNMISS alone group was 487.5 ml, while those with UNMISS with decompression surgery was 632 ml. The median operative time was 272.5 (202.5–335) minutes. The average operative time for UNMISS alone group was 242.5 min and the average operative time for UNMISS with decompressive surgery was 284.5 min.

### Post-operative complication

Two patients had wound dehiscence, which required debridement and re-suture wound. One patient developed worsened Frankel grading from E to D, which resulted from the further collapse of a non-operated segment (She underwent UNMISS from T1 to iliac and posterior decompression at T10 level due to high-grade metastatic epidural spinal cord compression). She underwent the second operation with extended spinal stabilization to C3 and decompression of the C5 level via the posterior approach. Motor power was fully recovered three weeks after the second operation. In patients with over one year of follow-up, there were no instances of construct failure. Additionally, there were no reports of medial or inferior breaches or screw malposition that required revision following the second intraoperative CT acquisition in any of our 14 cases.

### Treatment outcome

For the patients who received surgical treatment, ten patients underwent UNMISS with decompression surgery, and four patients required UNMISS alone. All patients had significant improvement in pain scores from 6.71 ± 2.20 to 0.29 ± 0.73 (*p* < 0.001). For the patients who received radiotherapy, all patients had significant improvement in pain scores from 7.66 ± 3.01 to 4.83 ± 2.14 (*p* = 0.013). However, the UNMISS group demonstrated significantly lower post-treatment VAS scores (*p* = 0.003).

In term of survival within UNMISS group, Five patients with Tomita scores ranging from 8–10 (predicted life expectancy of less than 3 month) [16] had a median survival time of 7 months. Seven patients with a Tomita score below 8 (predicted life expectancy of more than 16 months) [16] had a median survival time of 16.2 months. Two patients were lost to follow-up. 9 of 12 patients who had NESMS ranging from 0 to 2 (1-year postoperative survival rate of less than 50%) [14] died within one year. In the radiotherapy-alone group, 6 of 9 patients who had NESMS ranging from 0 to 2 (1-year postoperative survival rate of less than 50%) [14] survived after one year of treatment. However, there was no significant difference in the number of patient surviving equal or more than 12 months between groups (*p* = 0.306). The detail of treatment outcomes is shown in Table 4.

**Table 4:** Treatment outcome

Data	N (%) | Median (IQR) | Mean ± SD		*P*-value
	**UNMISS + RT (*N* = 14)**	**RT Alone (*N* = 9)**	
**Pre-treatment VAS**	6.71 ± 2.20	7.66 ± 3.01	0.439
**Post-operative VAS**	0.29 ± 0.73	4.83 ± 2.14	0.003*
**Mean differences in pre- and post- treatment VAS**	-6.429 95%CI ( -7.7, -5.1) *p* < 0.001	-2.833 95%CI ( -5.45, -0.22) *p* = 0.013	
**Type of surgery**			N/A
UNMISS with decompression	10 (71.42)		
UNMISS	4 (28.58)		
**Operative time (minutes)**	272.5 (202.5–335)		N/A
**Intraoperative blood loss (ml)**	475 (405–762.5)		N/A
**Post-operative complication**			N/A
Wound dehiscence	2 (14.3)		
Worsen neurological status	1 (7.15)		
**Survival time > 12 months**	5 (57.14)	6 (66.67)	0.306

## Discussion

This is the first case series of multiple spinal metastasis patients who underwent an ultra-long construct navigated minimally invasive spine surgery (UNMISS), which included the longest operated segment ever that had been reported. We found that both cohorts showed significant improvement in pain scores post-treatment (*p* = 0.01). However, the UNMISS group demonstrated significantly lower post-treatment VAS scores (*p* = 0.003), indicating enhanced pain relief. Survival outcomes did not differ significantly between the two groups.

Treatment of spinal metastasis patients requires a multidisciplinary approach. Surgical treatment remains the mainstay of stabilization of the spine and has evolved significantly over the recent years. Wagner et al. recently proposed a comprehensive algorithm for surgical management of patients diagnosed with spinal metastasis tailored to the patients' functional independence and disease. Any patient with symptoms that correspond with spinal metastasis was considered for posterior instrumentation and decompression first or other procedures in a selected case, such as adding anterior vertebral body replacement in a patient with good condition and slow-progressing systemic disease and osteolytic vertebral body, kyphoplasty in a patient who unfit for instrumentation and present with axial pain from the pathological fracture. Adjuvant radiation and systemic therapy should be offered to every patient [17].

Minimally invasive spinal stabilization (MISS) has been proven as the first-line surgical approach for spinal metastasis patients. Compared with conventional open surgery, MISS had a substantial advantage in terms of shorter length of stay, lower complications, less blood loss and transfusion rate [7], reduced postoperative opioid agents [9] and allowing earlier radiotherapy/chemotherapy [18]. The MISS technique also achieves a better quality of life, which relates to reduced VAS.

The most recent meta-analysis of David Eugenio Hinojosa-Gonzalez et al. on the comparison between open surgery and minimally invasive surgery(MIS) in spinal metastasis in 2022 found that, MIS appears to provide advantages over open surgery. This study analyzed 10 studies, totaling 577 patients, to determine the impact of MIS on spinal metastasis. MIS resulted in shorter length of stay (-3.08 days (95% CI, -4.50 to -1.66 days; *p* = 0.001)) and decreased odds of complications (OR = odds ratio of 0.60 (95% CI, 0.37 to 0.96; *p* = 0.03). However, the efficacy in pain reduction, and improving clinical grading score were similar to that of open surgery [19].

Long-construct percutaneous screw fixation was also previously defined with the insertion of percutaneous screw fixation on two vertebrae above and two below [10]. The ultra-long construct minimally invasive spinal stabilization was previously mentioned by Lee et al. [5]. They operated on the extensive spinal metastasis patient who had neurological deficit with the construct spanned over 15 spinal segments from T3 to L5 in combination with mini open direct decompression and vertebroplasty at L2. VAS was reduced from 6/10 to 2/10 at 6 weeks post-operation, and neurological improved from Frankel C to Frankel D. Patient passed away 7 months after surgery. However, the exact number of levels for ultra-long construct spinal fixation was not defined before. We defined the ultra-long construct navigated minimally invasive spine surgery (UNMISS) with the operated spinal segment of more than ten levels.

Regarding our results, all patients improved on their pain, and most patients can ambulate within ten days after surgery. Two patients had improvement in neurological function (from Frankel D to E). The addition of decompressive surgery did not significantly impact neurological improvement and pain, which correlated with the previous studies of MISS in spinal metastasis patients [6]. There has been no major postoperative complication in our case series, but the wound-dehiscence was encountered in two cases and worsened neurological status in one case. However, patients' quality of life, the durability of the ultra-long constructs without anterior reconstruction, and the safety and efficacy of adding decompression surgery in these patients should be further investigated in the following study.

Compared with the patients who received radiotherapy alone, the patients who underwent UNMISS had statistically significant lower post-treatment VAS (0.29 ± 0.73 for UNMISS and 4.83 ± 2.14 for RT alone, *p* = 0.003), but there has no statistically significant difference in survival period between group.

### Limitation

This study has several limitations that should be considered when interpreting the results. First, the small sample size, particularly in the radiotherapy-only group, limits the generalizability of our findings and may not fully represent the outcomes of a larger, more diverse population. Second, as a single-center study, the results may be influenced by institutional practices and the expertise of the surgical team, which might not be applicable to other settings. Additionally, the follow-up period was not uniform for all patients, potentially affecting the long-term outcome assessments.

Future studies with a prospective design, larger sample sizes, multicenter collaboration, and standardized treatment protocols are needed to confirm our findings and further evaluate the efficacy and safety of UNMISS in combination with radiotherapy for extensive spinal metastasis.

## Conclusion

UNMISS is a beneficial addition to standard radiotherapy for patients with extensive spinal metastasis. The primary goal of this technique is to stabilize the multiple levels of spinal metastasis and decompression of the neural element if needed. This technique is safe and has a better outcome in pain improvement than the patient who received radiotherapy alone.

## Data Availability

The datasets used and/or analysed during the current study available from the. corresponding author on reasonable request.
